# Measuring Fast-Temporal Sediment Fluxes with an Analogue Acoustic Sensor: A Wind Tunnel Study

**DOI:** 10.1371/journal.pone.0074007

**Published:** 2013-09-18

**Authors:** Ate Poortinga, Jan van Minnen, Joep Keijsers, Michel Riksen, Dirk Goossens, Manuel Seeger

**Affiliations:** 1 Soil Physics and Land Management Group, Wageningen University, Wageningen, The Netherlands; 2 Geography Research Group, KU Leuven Department of Earth and Environmental Sciences, Geo-Institute, Leuven, Belgium; 3 Department of Geoscience, University of Nevada Las Vegas, Las Vegas, Nevada, United States of America; 4 Physical Geography, University of Trier, Trier, Germany; Plymouth University, United Kingdom

## Abstract

In aeolian research, field measurements are important for studying complex wind-driven processes for land management evaluation and model validation. Consequently, there have been many devices developed, tested, and applied to investigate a range of aeolian-based phenomena. However, determining the most effective application and data analysis techniques is widely debated in the literature. Here we investigate the effectiveness of two different sediment traps (the BEST trap and the MWAC catcher) in measuring vertical sediment flux. The study was performed in a wind tunnel with sediment fluxes characterized using saltiphones. Contrary to most studies, we used the analogue output of five saltiphones mounted on top of each other to determine the total kinetic energy, which was then used to calculate aeolian sediment budgets. Absolute sediment losses during the experiments were determined using a balance located beneath the test tray. Test runs were conducted with different sand sizes and at different wind speeds. The efficiency of the two traps did not vary with the wind speed or sediment size but was affected by both the experimental setup (position of the lowest trap above the surface and number of traps in the saltation layer) and the technique used to calculate the sediment flux. Despite this, good agreement was found between sediment losses calculated from the saltiphone and those measured using the balance. The results of this study provide a framework for measuring sediment fluxes at small time resolution (seconds to milliseconds) in the field.

## Introduction

Quantitative evaluation of aeolian sediment fluxes is important to assess the varied roles of aeolian processes in landscape and nature development (e.g. [1]–[3]), in coastal defense (e.g. [4], [5]), and in nutrient dynamics especially in arid environments (e.g. [6]–[8]). Sediment fluxes are often measured using sediment catchers such as the Big Spring Number Eight (BSNE) [9], [10], the Basaran and Erpul Sediment Trap (BEST) [11], [12] or the Modified Wilson and Cooke sediment Catcher (MWAC) [8], [10], [13]. These traps are usually mounted in a vertical array to trap sediment at various heights above the surface. Sediment caught in the collection chamber is removed, dried, and weighed. By plotting the results as a function of height and fitting a curve through the data points the vertical sediment flux can be calculated. However, the data only provide information on sediment flux during the measurement interval itself. Moreover, there is no standardized method for the application of sediment traps and the data analysis method, which makes intercomparison between different studies difficult [14], [15].

The efficiency and behaviour of different sediment traps was reported in numerous studies [11], [13], [16]–[18]. Most of these studies used the controlled environment of a wind-tunnel, but some also performed a relative calibration in the field. However, due to the variety of techniques used when processing the data, the efficiencies reported were often not comparable. For example, for the MWAC sampler Sterk and Raats [16] using a three-parameter power function and a five-parameter combined model found an efficiency of between 43 and 66 %, whereas Goossens et al. [18] who directly compared the trap with an isokinetic sampler, reported efficiencies of 90 to 120 %. Mendez et al. [13] also found that the flux characterization used has a large impact on the calculated sediment flux.

A variety of instruments are currently available to investigate aeolian sediment fluxes over time [19], which can be grouped into four categories: (1) acoustic, (2) piezoelectric, (3) laser, and (4) pressure sensitive samplers. (1) The saltiphone [20] is a popular device, but other acoustic devices like loudspeakers [21] and small microphone systems [22] have also been used. Acoustic samplers register the signal generated when airborne particles strike a sensitive membrane. (2) The Sensit [23] and Safire [24] are examples of piezoelectric sensors. A small electric pulse is generated when a saltating particle hits a piezoelectric element. (3) The Wrenglor sampler is a laser-based system [25], [26] that uses a laserbeam and photo sensor to detect sediment particles. (4) Recently, a pressure sensitive sampler was developed and tested by Ridge et al. [27]. This instrument continuously monitors sediment accumulation by means of a water-level logger. However, it remains difficult to link the output of the instrument with the actual sediment budget.

Various studies [20], [28] have tried to directly link sediment fluxes measured by the saltiphone to actual sediment fluxes. However, none of these studies found an acceptable level of agreement. Reasons for this include: (1) the digital signal output used, (2) only one saltiphone was used, whereas data from different heights are required to characterize aeolian sediment fluxes for the entire sediment transport layer, and (3) the output of the saltiphone is only a representation of the amount of kinetic energy, which is difficult to directly link to sediment flux. Consequently, when using more than one saltiphone in an experimental array, all saltiphones need to be adequately calibrated as the response curve may slightly vary between instruments.

In this study, we test two passive traps (BEST sampler and MWAC sampler) and one acoustic device (saltiphone) in an aeolian sand wind tunnel to investigate how the experimental setup and the subsequent data processing affect the quantification of the aeolian sand flux.

## Materials and Methods

### Instrumental design

#### Modified Wilson and Cooke

**Figure 1:**
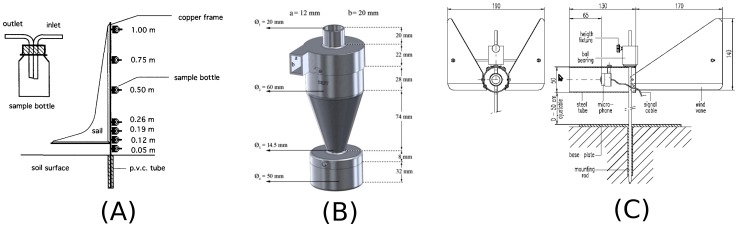
Schematic overview of the (a) Modified Wilson and Cook (MWAC) (from [16]), (b) Basaran and Erpul Sediment Trap (Best) (from [11]) and (c) the Saltiphone acoustic sampler.

The original Wilson and Cooke catcher (WAC) [29] consists of a bottle containing an inlet and outlet, whereby the trapped sediment is deposited in the bottle. In later studies, these bottles were mounted on a pole equipped with a sail to ensure that the inlet was always directed towards the wind (Fig 1a). This extended setup is called the Modified Wilson and Cooke (MWAC) trap. A detailed description of the conventional MWAC (referred to as MWAC-old hereafter) can be found in Sterk and Raats [16]. In the current study, we used a commercially available version of the MWAC, with an iron sail where the position of the bottles on the pole are adjusted (we refer to this modified setup as MWAC-new).

#### Basaran Erpul Sediment Trap (BEST)

The BEST, developed and tested by Basaran et al. [11], is a cyclone-type catcher with a conical shape (Fig 1b). Sediment enters the catcher via an inlet and follows a circular trajectory within the cone. The heaviest particles will settle due to gravitational and centrifugal forces whereas the lightest particles will be evacuated through the outlet. The principle is comparable to the separation of soil fractions in soil remediation equipment but BEST samplers are used with lower wind speeds to collect the smaller particles. Earlier developed cyclone samplers were mostly designed to measure dust (not sand) and may have similar conic shapes but were sometimes also cylindrical or elliptical [30]. Another difference between the BEST and the earlier developed cyclones samplers is that the BEST is composed of three parts instead of only one. The three units are: a lid including the inlet and outlet, a conical central body, and the proper collector.

#### Saltiphone

The saltiphone is a commercially available sampler which consists of a microphone installed in a stainless steel tube mounted on a ball bearing (Fig 1c). Two vanes at the back of the tube ensure proper alignment with the wind. The ball bearing can be connected to a stain rod, which is height-adjustable. A cable connects the microphone to the electronics, which is stored in a waterproof aluminium housing. Sand particles that hit the microphone produce a high-frequency signal. Frequencies of about 8 Khz are amplified and used to determine saltation whereas other frequencies that are caused by rain and wind are reduced using a narrow band filter. The pulse created by each particle is cut off after 1 millisecond. Two output signals are provided: a digital pulse and an analogue voltage. The digital signal gives an output that is translated into number of counts. The analogue output signal also provides this information but has the additional option of measuring the intensity of particle impacts because it measures the energy of impact on the membrane. In this mode, the output signal represents the kinetic energy of the particles, and thus particle size and speed. The analogue output option was used in this study. Data were measured with the same interval as the sampling rate of 1 millisecond.

### Experimental setup

**Figure 2:**
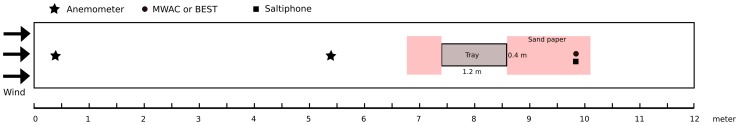
Schematic (top view) diagram the experimental setup. A balance was placed underneath the test tray to measure the weight of the sediment throughout the experiment.

The study was conducted at the wind tunnel of the International Center for Eremology (ICE), Ghent University, Belgium. The wind tunnel has a length of 12****m and is 1.2****m wide and 3.2****meters high [31], [32]. Wooden spires and roughness cubes were placed to create a boundary layer of 0.6****m at the entrance of working section of the wind tunnel [31]. A test tray of 1.2****m long, 0.4****m wide and 0.012****m deep was placed at 7.4****m downwind from the entrance and filled with sand (Fig 2). To ensure similar roughness compared to the sand, sand paper was applied before and after the tray. Wind velocity was measured using five vane-type probes (type 0635

9540, Testo GmbH & Co, Lenzkirch, Germany). These probes have a vane diameter of 16****mm and are appropriate to measure wind velocities up to 60****ms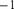. The first was installed at 70****cm height near the upwind edge of the test section and the others 2.1****m in front of the tray at 5, 10, 15 and 30****cm heights, respectively. Wind velocities were measured with one-second intervals. The sediment catchers and saltiphones were installed downwind from the test tray and were separated by a distance of 10 cm (Fig 2).

**Figure 3:**
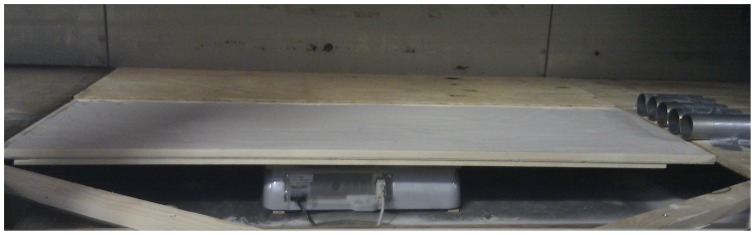
Image of the experimental setup. The balance was placed underneath the test tray in order to measure the weight of the sediment throughout the experiment. The image taken during the calibration, when the five saltiphones were placed next to each other.

To measure the sediment loss during an experiment, a balance was placed underneath the test tray (Fig 3). The balance was programmed to register the time when the weight of tray changed. However, during an experiment the air pressure can change thereby potentially affecting the measurements. Therefore, several test runs were performed with a fully covered tray. Results indicated that any potential effects were within the measuring error of the balance. Thus, corrections for pressure differences were not required in this study.

### Sediment

**Figure 4:**
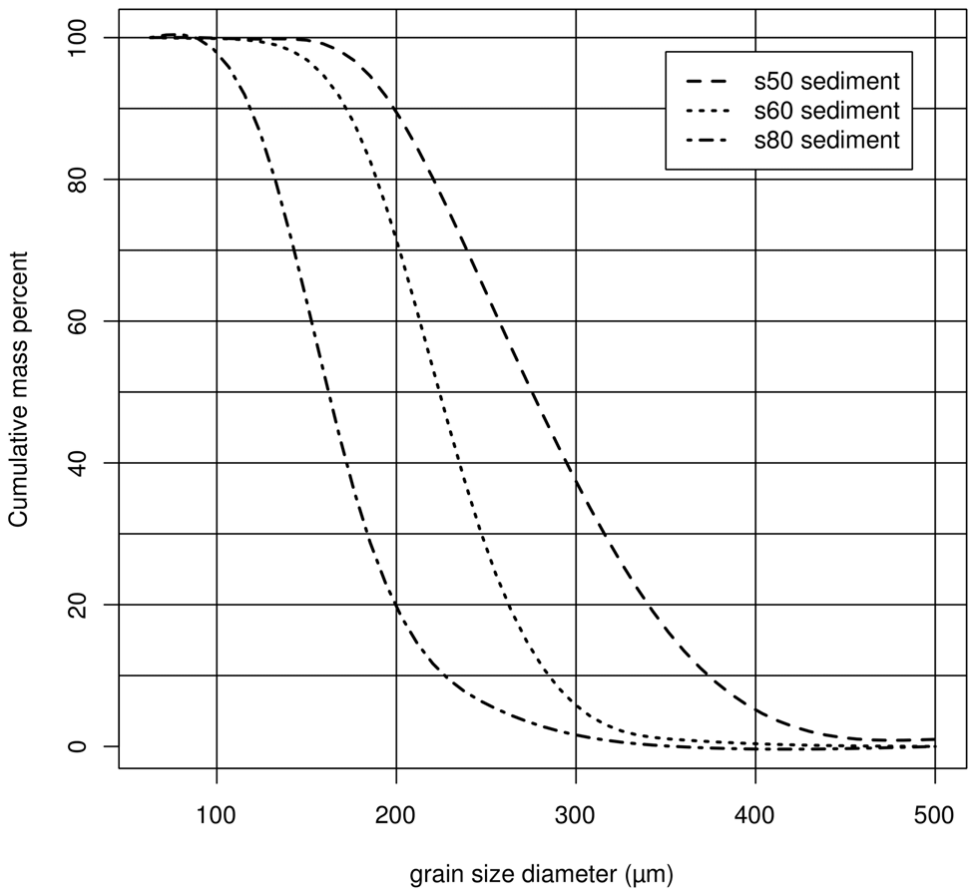
Grain size distribution of the three sediment types (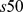, 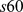 and 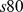) used in the wind tunnel experiments.

Three industrial sands (referred to as 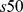, 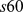 and 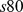) were used. All sediments consisted predominantly of quartz (99.5%) with traces of hematite, aluminium oxide and titanium dioxide. All sands were industrially washed and pre-sieved. The median diameters (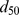) were 285, 230, and 170**** *µ*m, respectively, with their grain size distributions shown in figure 4.

### Analysis method

#### Wind Data

Wind data collected from four altitudes were used to calculate the roughness length (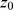) and shear velocity (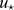) using the law of the wall:
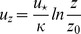
(1)where 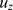 is the wind speed at elevation 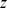 above the bed, 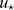 is the shear velocity, and 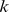 von Karmans constant (0.4). Plotting the elevation on a vertical axis and the wind speed on a horizontal axis, rearranging equation 1 into 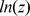  =  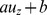 , and applying a regression analysis, the values of 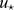 and 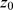 were calculated as 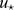  =  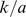 and 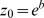, respectively.

The threshold shear velocity was calculated using equation 2 [33]:
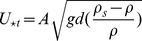
(2)where 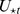 is the threshold shear velocity, 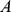 is a dimensionless constant (assumed to be 0.085 for the fluid threshold and 0.1 for the impact threshold), 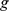 is the gravitational acceleration (ms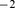), 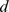 is median (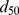) grain size (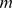), 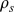 is the density of the sediment (kgm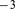) and 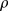 is the density of air (kg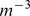).

#### Saltiphone

**Figure 5:**
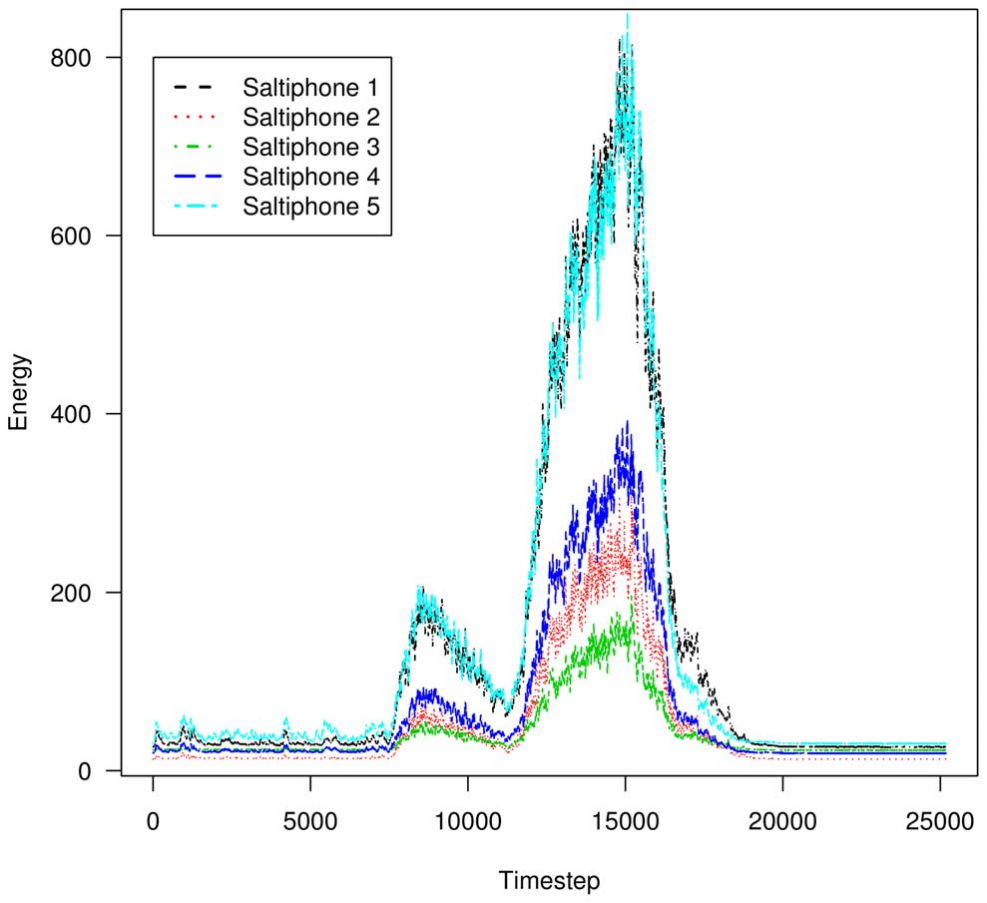
Output signal of the five saltiphones when placed next to each other. Output represents energy but is dimensionless.

For a given impact, the analogue energy output signal may vary between saltiphones. Therefore, a calibration procedure was developed, where all five saltiphones were deployed next to each other (Fig 3). Figure 5 shows the raw output signal of the saltiphones placed horizontally next to each other under constant saltation conditions. Two observations were made. (1) During periods without saltation there is still a signal because in the analogue energy mode, the output signal is sensitive to the input signal (volts) and, (2) the amplitude of the output is different for the different saltiphones, even when sediment transport is measured under similar conditions. This problem can be resolved by using one saltiphone as a reference, because the temporal patterns of the output signals are very comparable (Fig 5). In this study, the saltiphone in the centre was used as the reference. Before and after the experimental runs, the output of each saltiphone was recalculated using a simple linear regression 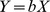 where 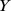 is the output of a given saltiphone and 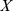 the output of the reference saltiphone. To account for horizontal variability in sediment flux, the positions of the saltiphones were regularly changed during the calibration.

#### Regression Analysis

For each trap in the vertical array the total amount of sediment caught was multiplied by the area of the inlet to get an amount in kgm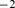. These data were used for regression analysis to calculate the vertical transport flux within the entire sediment transport layer. However, there is disagreement in the literature as to how to best describe the vertical profile of sediment transport [14] [34]. Exponential functions (equation 3) as well as power function (equation 4) have been used:



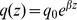
(3)

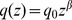
(4)


The regression parameter 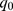 is often associated with the portion of creep, whereas 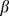 represents the decay rate with height 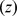. To facilitate calculating this regression, some software packages use these formulae in a logarithmic form [35]:
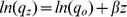
(5)

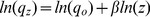
(6)


Note that the result for 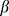 will be different for these two approaches because of the difference in the last term on the right.

**Figure 6:**
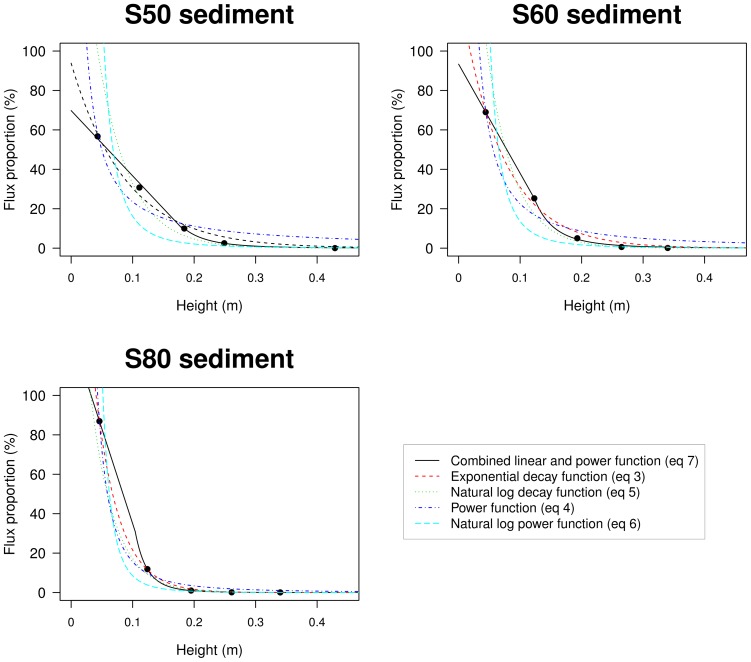
Five regression models plotted through the data points, for the three sediments tested (s50, s60 and s80).



Figure 6 shows the relative (normalized) sediment flux plotted against height for three representative runs of each test sediment. The data were taken from three measurements with the MWAC-old, where the sediment from the bottles is expressed as a portion of the total sediment flux in order to make them comparable. The data indicate that neither the power nor the exponential function adequately describe the measured sediment profiles of the s50 and s60 sediments. Visual interpretation of the profiles suggests a linear trend in the lowermost part of the saltation layer. For the s50 sediment, a linear line can be fitted through the three measurements points closest to the bed. Whereas for the s60 and s80 a linear line can be drawn to the two points closest to the bed in the upper part of the profile a power function gives the best fit. Therefore the following combination was used: a linear function in the lowermost part of the saltation layer and a power function in the upper part. Separate regressions were made for each part and the total sediment flux (kgm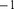) is calculated as follows:

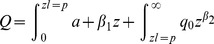
(7)The two functions intersect at the point 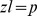, where 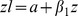 and 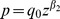.

#### Sediment Fluxes

**Figure 7:**
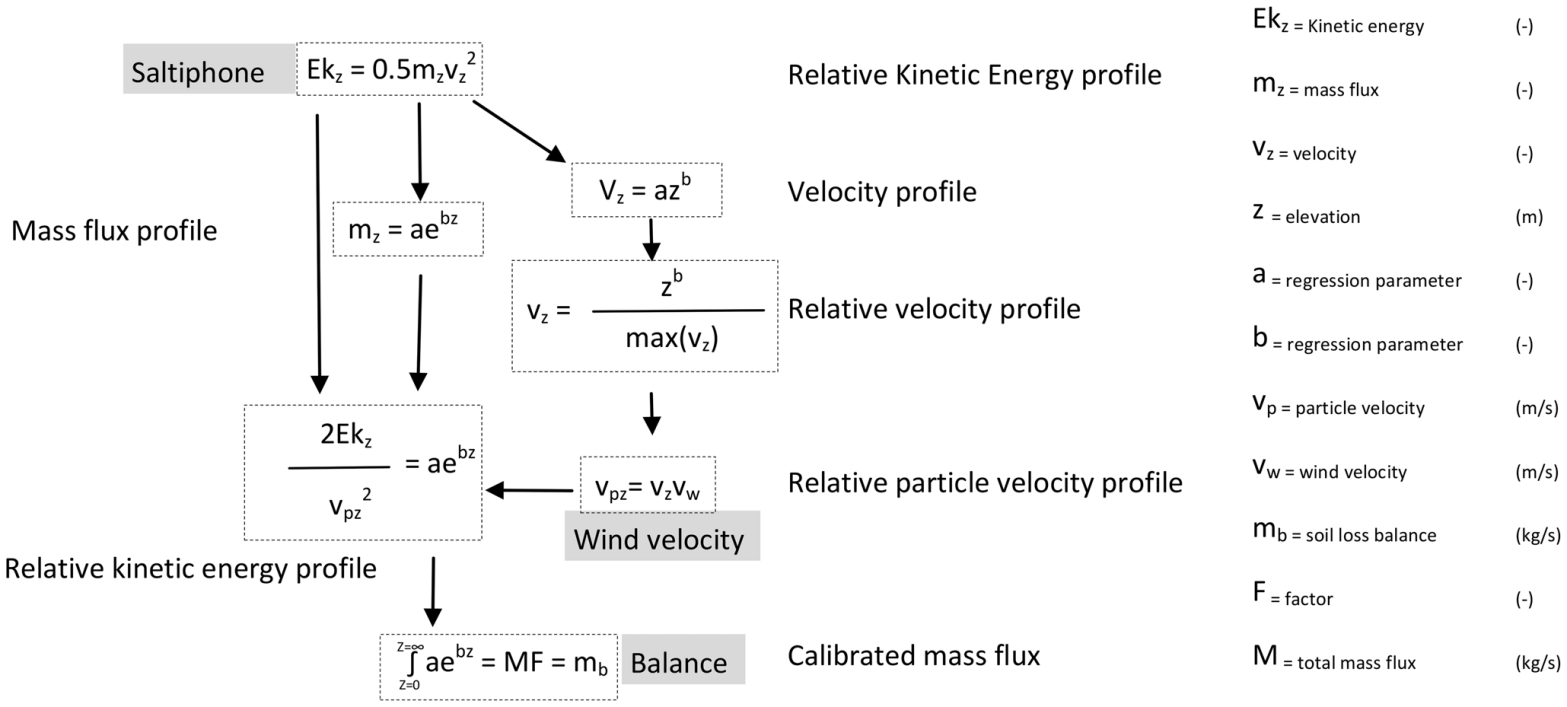
Sediment fluxes were calculated from the saltiphone data using wind data and data from the balance.

Sediment fluxes were calculated by combining the saltiphone data with wind speed data and data from the balance. Figure 7 presents a schematic overview of the procedure.

The amplitude of the analogue output of the saltiphone is determined by the kinetic energy of the particles hitting the membrane of the microphone. This kinetic energy depends on the mass (kg) and velocity (ms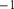) of the particles (equation 8):
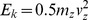
(8)


However, the translation of vibrations of the membrane to a voltage is influenced by the characteristics of the membrane. Therefore, the analogue output cannot be directly translated to Joule (J), the unit of kinetic energy. Moreover, no data on particle velocity were collected during the experiments. Equation 8 can thus only be used to express the characteristics of the sediment flux in relative terms.

To estimate the variation of kinetic energy of the impacting particles with height, we used separate functions for the particles' mass and velocity. For the mass, we used an exponential function similar to equation 3, and for velocity, a power function similar to equation 4. The exponential function was chosen because experimental work has shown that the vertical distribution of the sand transport rate of medium and fine-grained sands (such as the ones used in this study) is typically expressed by such a function [36]. The power function was selected based on the studies [37], [38]. The variation in kinetic energy with height is then described by:



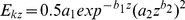
(9)
Equations 8 and 9 were used to estimate particle velocity during the experiments using the following steps. First, the total analogue output for all five saltiphones together was calculated. Next the relative proportion of each saltiphone in the total analogue output was computed. The measured sediment fluxes of the sediment traps were treated in the same way to calculate the relative proportion of each trap in the vertical array. The saltiphone and sediment trap data were then correlated in a non-linear model, where the parameters 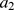 and 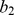 were optimized until a weighted least-squared optimum was found. The value derived for 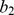 was used to estimate an average particle velocity at the elevation of the saltiphone. As this can only be done in relative terms, the particle velocity (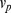) profile was fitted for a 30-cm interval using 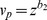 (see equation 9) and normalized by dividing it through the maximum value of 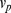. To estimate the real particle velocity the relative particle velocities were then multiplied by the wind velocity of the highest anemometer (30****cm), which is located close to the sediment tray.

All saltiphone data (which were measured every millisecond) were averaged to seconds to ensure the same temporal resolution as for the wind data. Particle velocity was then calculated for every second. From the 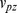 and 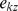, the relative mass flux at the elevation of the saltiphone was calculated using equation 8. To obtain the total sediment flux an exponential function (equation 3) was fitted through the data points. Integration over the entire height of the sand transport layer then yielded the total sediment flux. However, because 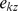 is dimensionless and 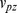 is described as a function multiplied by the wind velocity, the total sediment flux measured by the saltiphones was compared with the total amount of soil loss measured by the balance. A linear function (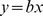) was fitted through the data points to scale the relative output of the saltiphone (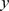) to the balance (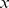). The value of 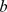 was then used to convert the calculated sediment flux from the saltiphone into a real sediment flux. This was done by using equation 10, which is equation 8 with the inclusion of a factor 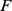 and 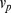 as particle velocity. The 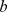 value found in the linear regression was used as 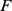 in equation 10.
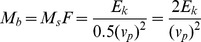
(10)

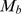
 represents the mass flux from the balance, and 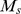 the flux measured by the saltiphone.

### Efficiency

In this study efficiency is defined as the ratio of the vertically integrated (over the entire height of the sand transport layer) sediment flux as measured by the catcher, relative to the total sediment flux derived from the sediment loss from the balance. The vertical integration can be done using any of the empirical approaches displayed in the equations 3 to 7.

## Results and Discussion

### The efficiency of the different catchers

**Figure 8:**
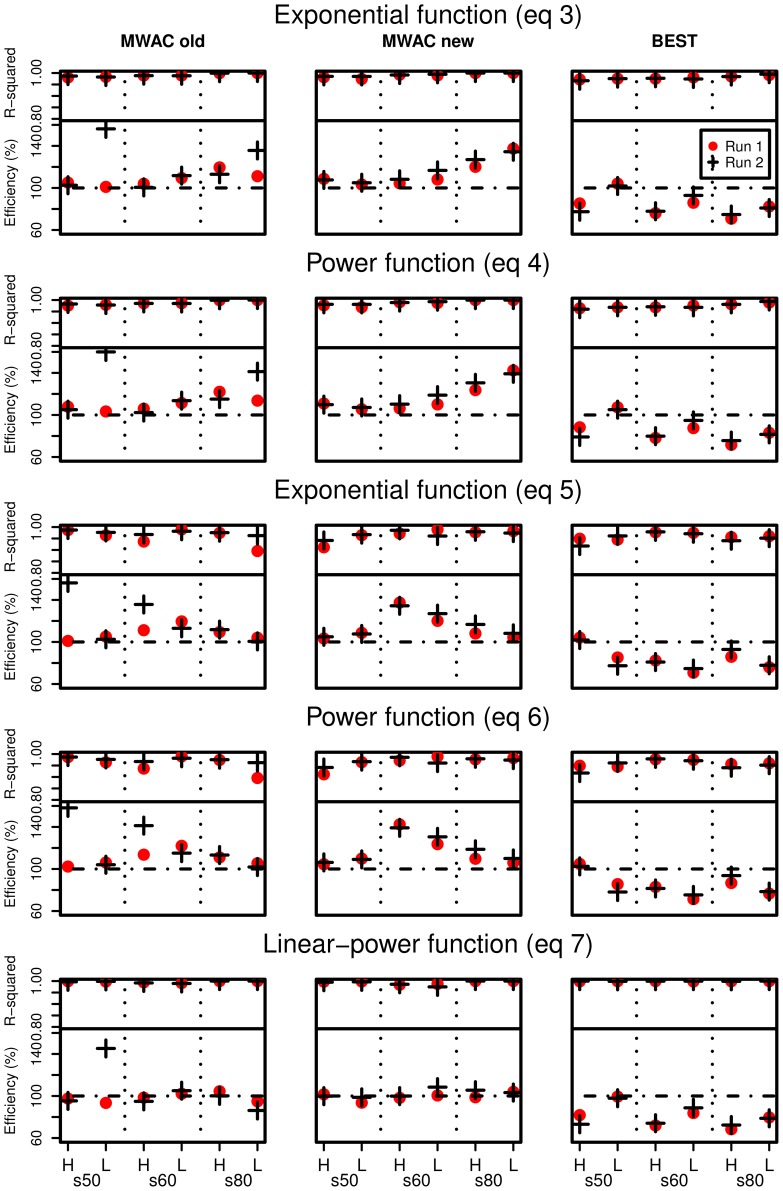
Efficiencies and goodness of fit (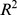) of the three catchers as calculated from five regression models. H and L are high and low wind velocity, respectively, and s50, s60 and s80 are the three sediments used in this study.

Efficiencies were calculated for all five approaches (equations 3 to 7), with the results shown in Figure****8. The ordinate displays the calculated efficiencies (%) as well as the goodness of the statistical fit for each approach (using 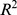) with the results being very dependent on the equation (approach) used. A similar conclusion was made by Panebianco et al. [34]. For both MWACs, the combined linear-power equation gives the best results, with efficiencies around 100%. For the BEST sampler, the exponential function (equation 3), the power function (equation 4) and the combined linear-power function gave similar results, with efficiencies around 80%. The importance of the statistical software package used can also be seen: for the same experiment, large differences in calculated efficiencies may be obtained depending on whether or not the logarithmic versions (5) and (6) of equations (3) and (4) were used. The logarithmic versions also resulted in a poorer fit (lower values for 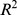).

**Figure 9:**
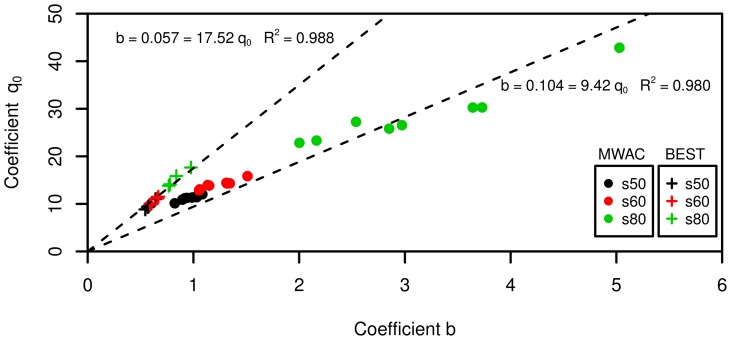
Dimensionless regression coefficients 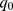 and 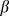 calculated for the MWAC and BEST samplers, for the three sediments used in this study.

The difference in efficiency between the MWAC and the BEST when using the exponential function is most probably explained by the elevation of the lowest trap. For the BEST, the lowest trap was located directly on the surface, whereas for the MWAC, the lowest bottle was located around 4–5****cm from the surface. When an exponential curve is fitted through the data points, the 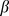-exponent is mainly determined by the slope between the two lowest points. The higher these points are situated above the surface, the more likely 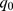 and 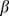 will become overestimated. This can also be seen when 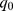 and 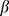 are calculated for the normalized sediment flux (the amount of sand captured in a bottle relative to the total amount in all the bottles). Figure 9 illustrates this overestimation. Literature [39], [40] shows that a perfect linear relationship between 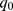 and 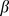 can be expected under similar conditions of surface moisture and sediment. In Figure 8 the relationship is excellent for the BEST catcher whereas it is less pronounced (but still remains acceptable) for the MWAC catcher. The lower correlation and the different value for the slope for the MWAC are likely caused by the higher position of the lowest bottle, resulting in a larger uncertainty for the flux in the lowermost zone of the sediment transport layer and an overestimation of the values for 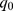 and 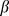. When equation 7 is used, efficiency is mostly around 100% (Fig. 8), suggesting that the exponential function overestimates 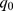 and 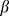. The results for the BEST sampler, point towards the same conclusions since equations 3 and 7 produce similar results.

No relationships were found between efficiency and sediment type and efficiency and wind speed. This contrasts with previous results [17], where higher efficiencies occurred as the sediment became coarser, and where differences in efficiencies as the wind speed increased were also noted. However, in [17] a large range of sediment sizes was used, varying between 50–500**** *µ*m. The result here are in agreement with other studies [16], [18], who found no relation between the efficiency of the MWAC catcher and wind velocity. MWAC efficiency is substantially determined by the experimental setup (in particular, the elevation of the lowest bottle) and the analysis method (type of regression) used. The current study suggests that efficiencies close to 100% results when exponential curve fitting is used. For the BEST sampler, almost identical efficiencies were observed regardless of the curve fitting technique used.

### Calibration of the saltiphones

The saltiphones were calibrated before, during and after the experiments. In total, 12 calibration experiments were performed, where the energy output of the saltiphone in the center (saltiphone 3, see Fig. 5) was used as the reference. The duration of a calibration run was approximately 3–4 minutes. To avoid results being affected by potential differences in sediment concentration across the wind tunnel's test section, we reversed the relative position of the saltiphones during several of the tests (saltiphone 1 was moved to position 5 and saltiphone 5 to position 1; and saltiphone 2 was moved to position 4 and saltiphone 4 to position 2; saltiphone 3 remained in place at all times) and averaged the result. To estimate the difference in sediment concentration between position 1 and position 5, the difference in energy output between saltiphones 1 and 5 was compared for the two setups and the average was calculated; the same procedure was followed for saltiphones 2 and 4. As expected, sediment concentration was not identical within the wind tunnel section. At position 1 (Fig. 5, saltiphone in the back) it was 48% higher than in the center, and at position 2 it was 23% higher; at position 4 it was 22% lower than in the center, and at position 5 (Fig. 5, saltiphone in the front) it was 37% lower. This difference in horizontal sediment flux was incorporated into the output data of the saltiphone. With this correction, the calibration factor (i.e. the difference in response between the saltiphones) was calculated (Table 1).

**Table 1:** Calibration of the Saltiphones using linear regression (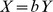).

Saltiphone number	Calibration factor *b*
1	2.4
2	1.9
3	1.0
4	2.8
5	1.6

Parameter 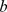 expresses the multiplication factor of the representative saltiphone to saltiphone 3.

The variation in sediment flux over the tunnel section is rather large considering the relatively homogeneous wind field in the test area [31]. Basaran et al. [11] used a transparent sellotape to determine this variation for different sediments and wind velocities in the wind tunnel used in the current study and found that 29.7 to 55.5% of the sediment was transported within the central 35 cm of the tunnel section.

### Sediment fluxes calculated from the saltiphone

Sediment fluxes were calculated for every second, based on the kinetic energy measured by the saltiphones. This was done in three steps: (1) fitting a function through the individual data points to establish the kinetic energy profile, (2) determine the particle velocity profile, and (3) calculate the sediment flux from the kinetic energy and particle velocity profiles.

### Kinetic energy

**Figure 10:**
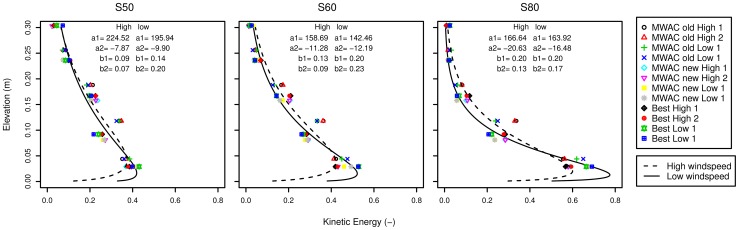
Normalized kinetic energy flux for the three sediments, for high and low wind velocities. 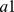
, 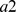, 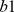 and 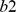 are the regression parameters from equation 9.

Figure 10 shows the normalized kinetic energy plotted against elevation for the three sediments tested. The values for 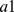, 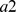, 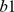 and 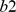 are also shown.

A peak occurs in the normalized kinetic energy around 2 cm above the surface for all three sediments. This peak is more pronounced as the sediment becomes finer. Therefore, for fine sediments, a larger fraction of the kinetic energy is found close to the surface compared to coarse sediments. For the latter, the total kinetic energy carried by the airborne particles is less concentrated near the bed. These results are consistent with previous findings [38].

### Particle velocity profile

The particle velocity profile can be constructed using the power function 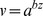 [37] [38]. Note that the value for 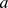 depends on the choice of the units; by normalizing the particle velocities, the exponent 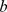 fully describes the profile.

**Figure 11:**
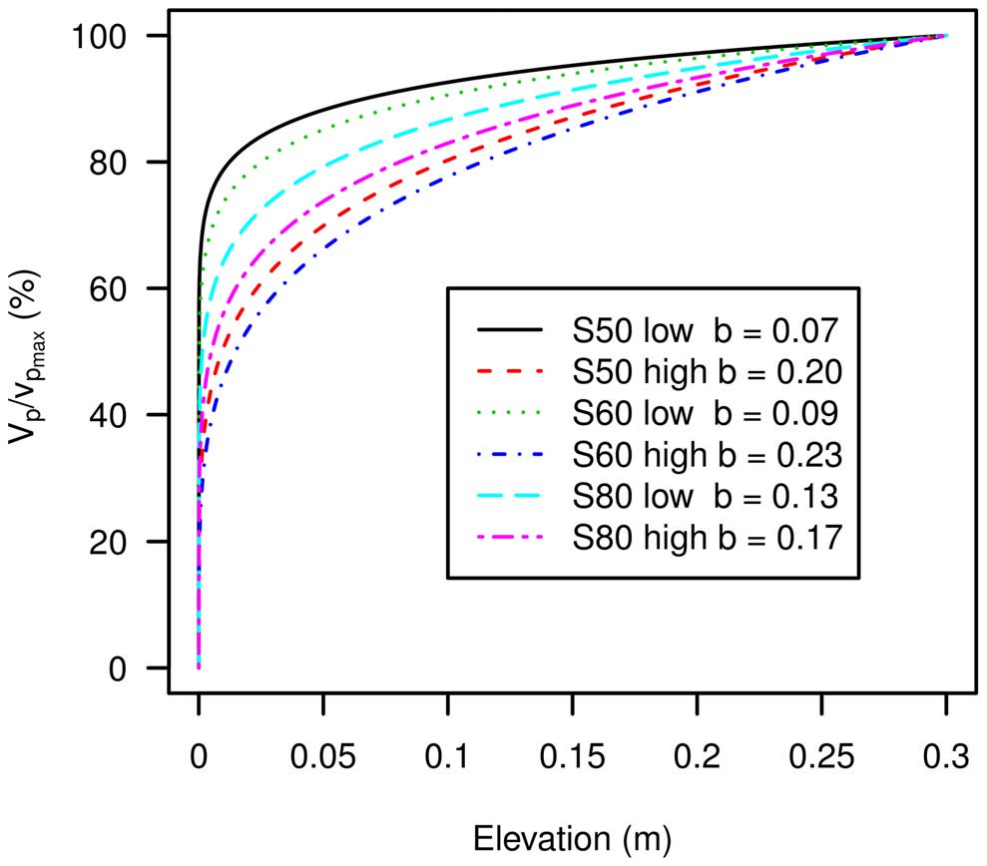
Particle velocity profiles for the three sediment types used, for high and low wind speed.

Figure 11 shows the normalized profiles. For high wind velocities the value for 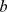 increases from 0.07 for the coarsest sediment (s50) to 0.17 for the finest sediment (s80). For low wind velocities the *b*-values are 0.20 (coarse sediment s50), 0.23 (medium-sized sediment s60), and 0.17 (fine sediment s80).

The normalized velocity profiles differ for the two wind speeds investigated. Therefore, we opted for using the average of both wind speeds when calculating the particle velocity profile for the whole experiment.

### Sediment fluxes

**Figure 12:**
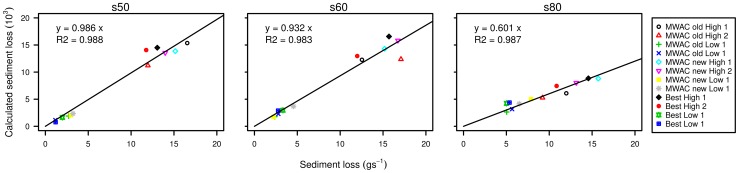
Sediment loss measured from the balance compared to the total sediment loss calculated from the saltiphones, for the three sediments tested in this study.

In Figure 12, we compare the calculated total sediment flux with the measured soil loss from the balance. Good relationships were found between the measured and calculated flux for all three sediment types, but the slopes of the curves differ. For the coarse (s50) and medium-sized (s60) sediment the F-value was close to unity (0.986 and 0.933, respectively), whereas for fine sediment (s80) the F-value was 0.601.

Previous studies [39], [41] have shown that particle velocity decreases with an increase in particle size. For the current study, this would imply that the particles of sediment 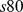 should have higher velocities compared to those of sediments 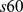 and 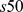. Rearranging equation 10 into:
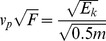
(11)and using the F-values derived from Figure 11, our results confirm this trend. For a given amount of kinetic energy and a specified amount of mass equation 11 predicts a lower particle velocity as particles become coarser. However, no direct measurements were made of the particle velocity in this study. Also, the physical characteristics of the saltating grains were not taken into consideration. In reality, the sediment source is characterized by a mix of different shapes and sizes, and every particle will have its own saltation trajectory. The angle at which the particle hits the microphone might also have a considerable impact on the total amount of energy transferred to the membrane.

**Figure 13:**
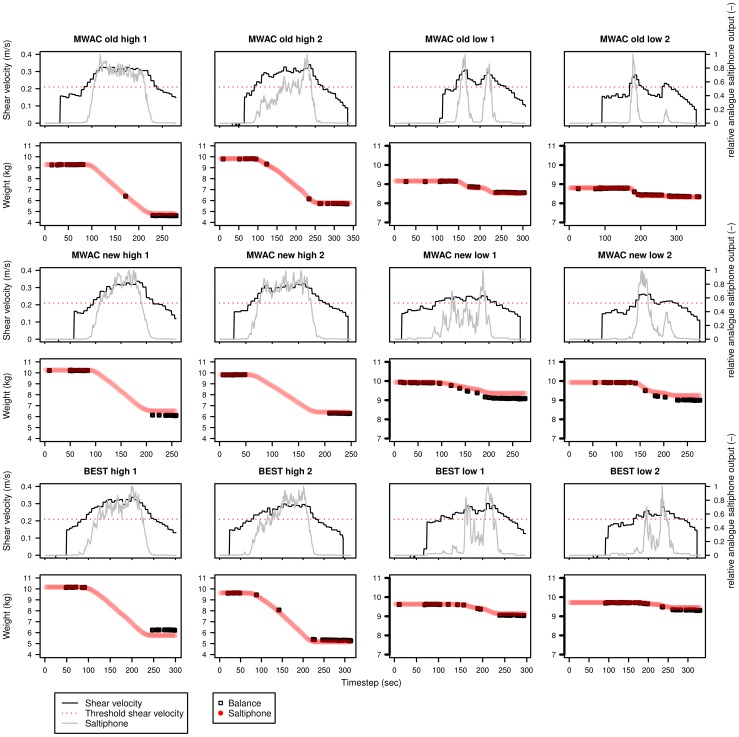
Sediment loss, relative analogue saltiphone output and shear velocity over time for the different experiments with sediment s50. The experiments were done using three types of catchers (MWAC-old, MWAC-new and BEST) with high and low wind velocities. Two replicates were done for each test. Sediment loss was measured from the balance (black) and calculated from the saltiphone (red).

**Figure 14:**
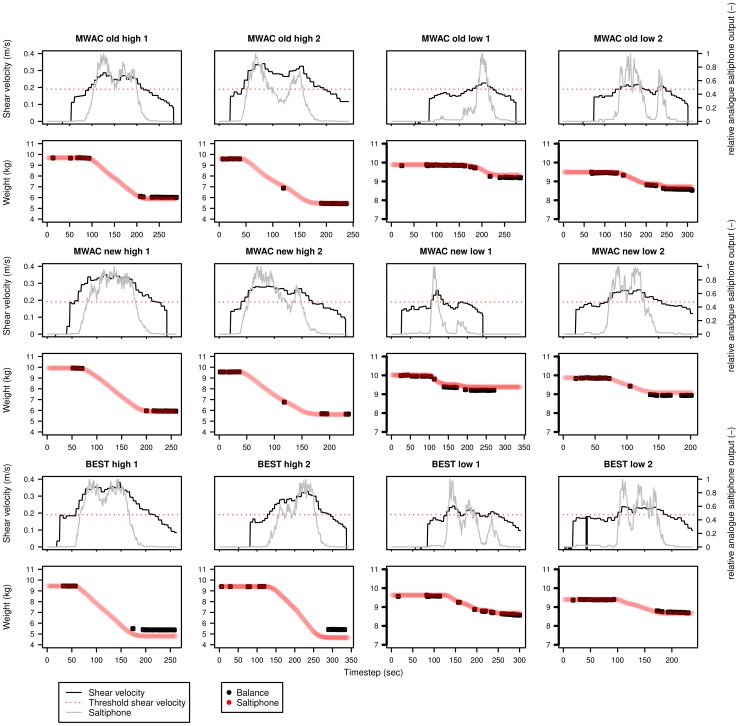
Sediment loss, relative analogue saltiphone output and shear velocity over time for the different experiments with sediment s50. The experiments were done using three types of catchers (MWAC-old, MWAC-new and BEST) with high and low wind velocities. Two replicates were done for each test. Sediment loss was measured from the balance (black) and calculated from the saltiphone (red).

**Figure 15:**
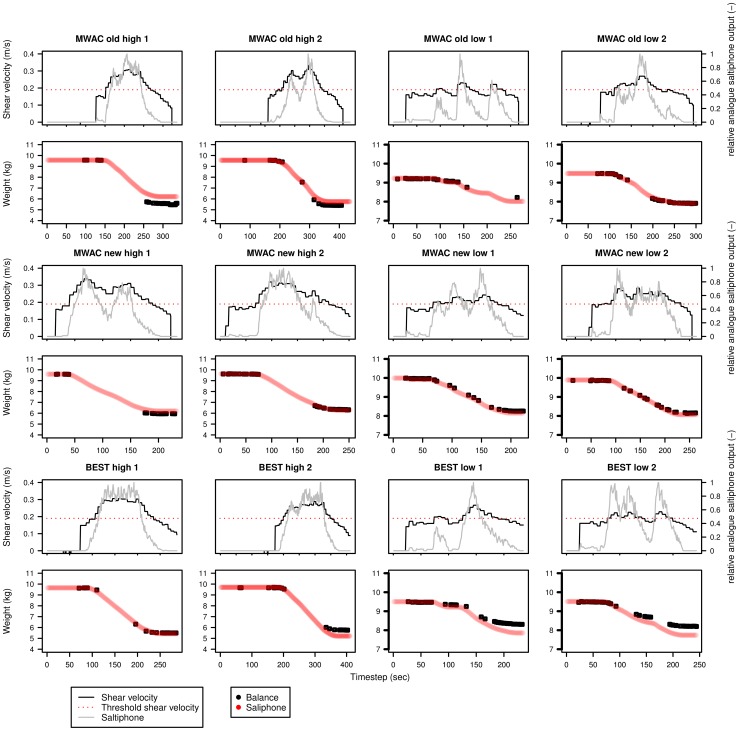
Sediment loss, relative analogue saltiphone output and shear velocity over time for the different experiments with sediment s50. The experiments were done using three types of catchers (MWAC-old, MWAC-new and BEST) with high and low wind velocities. Two replicates were done for each test. Sediment loss was measured from the balance (black) and calculated from the saltiphone (red).

Equation 11 was used to calculate the sediment flux with an exponential function fitted through the data points to estimate the total sediment flux for each second. Results are displayed in Figures 13, 14, 15 for sediments s50, s60 and s80 respectively. Each sediment type has a total of 12 experiments, for three different sediment catchers (MWAC-old, new and BEST) using high and low wind velocities in duplicates. Each figure shows the shear velocity, threshold shear velocity and normalized analogue output of the saltiphone. The normalized output was calculated by summing all calibrated outputs of the saltiphone and divide this sum by the maximum value during one experiment. Also shown in Figures 13, 14, 15 are the output (weight loss) recorded by the balance and the sediment flux calculated from the saltiphone data.

Results show that the analogue output of the saltiphone can indeed be used to assess sediment fluxes on a small temporal time scale. For the s50 sediment, the results for the measurements with the new MWAC at the lowest wind velocity show a small underestimation, whereas the BEST gives a small overestimation for the highest wind speed. The same is true for the s60 sediment, but for the s80 sediment, an over estimation can be seen for the second run, for both wind speeds. Accepting a small measurement error in the balance weights all results are well within acceptable boundaries.

**Figure 16:**
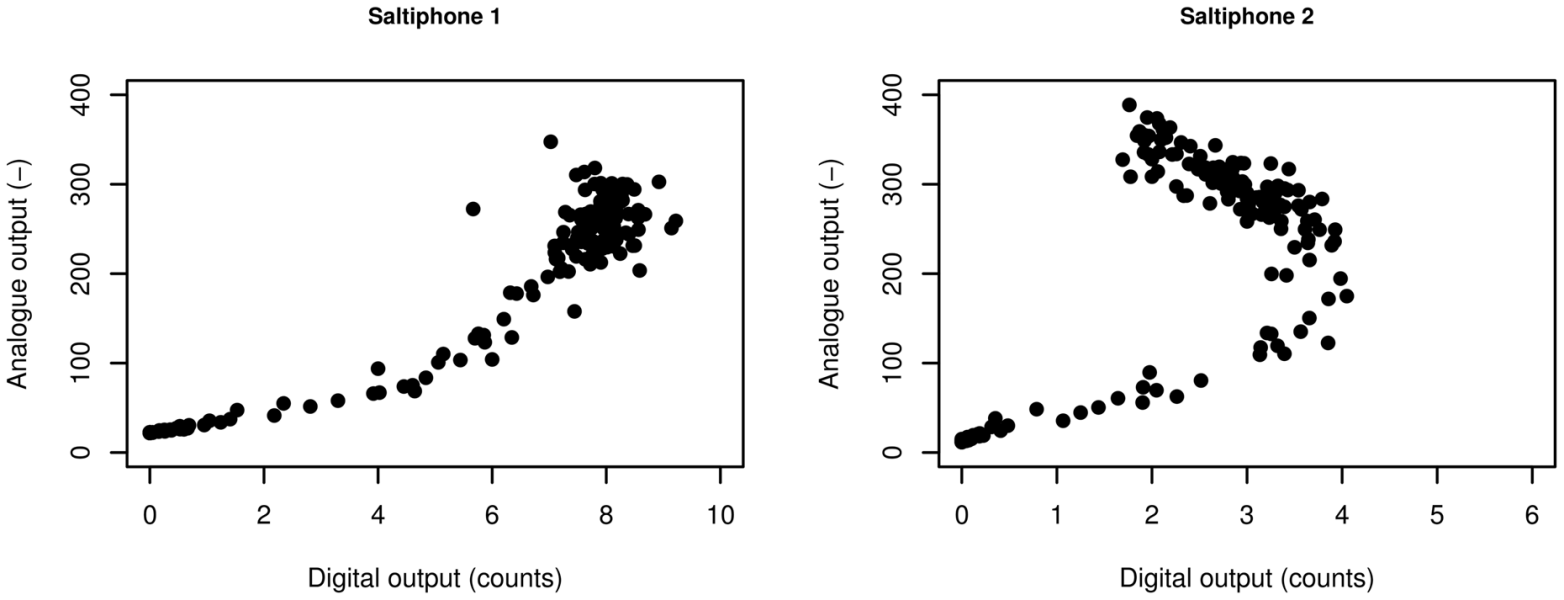
Raw analogue output compared to the raw digital output for the first two saltiphones.

To check whether or not the procedure to calculate the sediment flux from the analogue output of the saltiphone can be replicated by using the saltiphone's digital pulse output, the two raw signals were compared. Figure 16 shows the results for the first two saltiphones. For saltiphone 1, there is a good correlation between the two outputs, but at high energy levels the relationship becomes less well expressed. The output of saltiphone 2 illustrates why the digital pulse output cannot be used to quantitatively assess wind erosion as an almost parabolic relationship was found between the digital pulse and the analogue output. Saturation might be the most plausible cause for this phenomenon.

### Shear velocity and sediment fluxes

Apart from sediment flux, Figures 13, 14 and 15 also show both shear velocity and threshold shear velocity. Shear velocities and roughness lengths (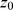) were calculated from the wind velocity profiles. Data for the roughness length varied considerably, from 0.002 to 0.103****mm (a factor of 50). These values are low compared to the values measured for comparable sands (refer to [42]), who used a value of 1****mm. Threshold shear velocities were calculated for all three test sediments by means of equation 1, using the median grain diameter (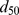) as the reference diameter and using particle density equal to 2650****kg 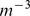.

**Figure 17:**
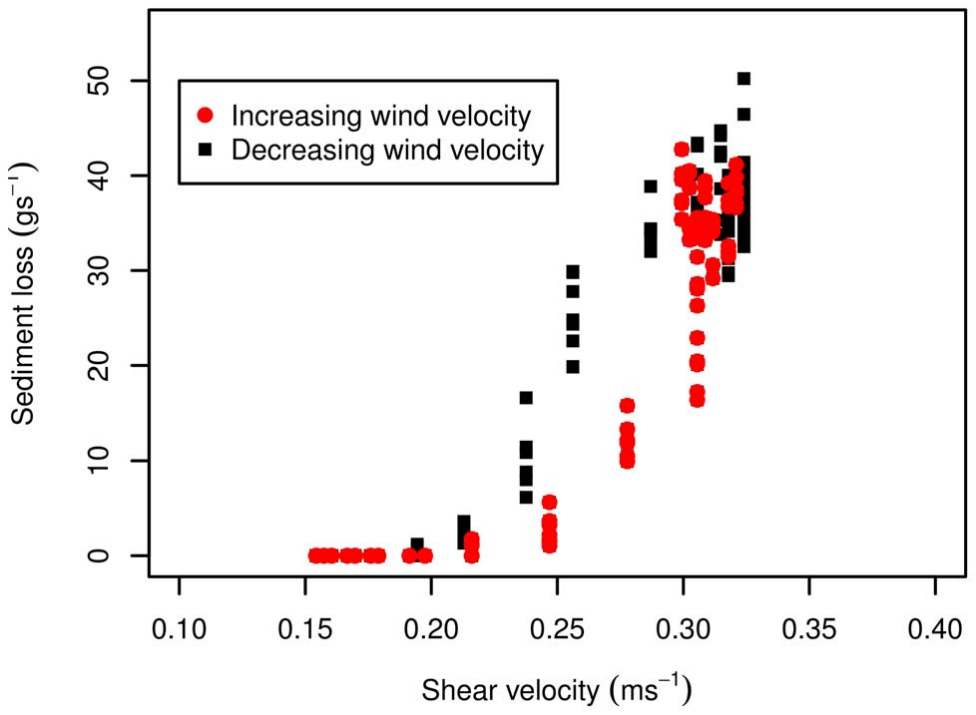
Sediment flux for different shear velocities, for an accelerating and a decelerating wind velocity.

The calculated threshold shear velocities (table 2) are consistent with the data. When shear velocity exceeds the fluid threshold sediment transport is measured by the saltiphone. However, the data also show a clear difference between high energy levels and low energy levels (Fig. 17). When wind is still accelerating, sediment fluxes are lower than when the wind is slowing down. This phenomena is also known as hysteresis, which means sediment flux is not only dependent on the current shear velocity, but also on the previous shear velocities [43].

**Table 2:** Threshold shear velocities calculated for the different sediments using equation 2.

sediment	impact threshold U_*t_ ms^−1^	fluid threshold U_*t_ ms^−1^
s50	0.21	0.25
s60	0.19	0.22
s80	0.16	0.19

### Comparison between the saltiphone and the BEST trap

To determine whether the technique developed in this study to calculate sediment fluxes from saltiphone data leads to more accurate results, the sediment flux profiles from the saltiphone and the BEST sampler were compared. For the saltiphone we first calculated the average fluxes of the individual experiments. Relative fluxes were then calculated by dividing the sediment flux obtained from each saltiphone by the total of all saltiphones. The same procedure was adopted for the BEST. Only the BEST was used in the test because this sampler provides more data points in the saltation layer than the MWAC, which guarantees a better characterization of the sediment flux profile.

**Figure 18:**
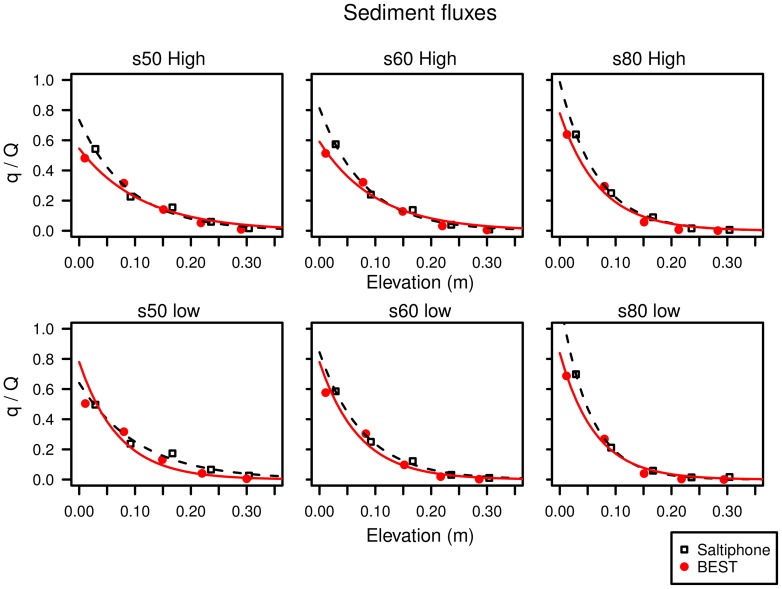
The total dimensionless sediment fluxes of the BEST and Saltiphone compared. s50, s60 and s80 are the three sediment types tested; High and Low refers to high and low wind velocity.

Results are shown in Figure 18 with an exponential function used to fit the data points. Similar patterns were obtained for all wind speeds and sediments tested. In general, the results are comparable for the saltiphone and the BEST, illustrating the usefulness of the techniques. For the two coarsest sediments (s50 and s60), the agreement is less encouraging close to the bed for the high-wind velocity case. At low elevations, the saltiphone overestimates the sediment flux compared to the BEST.

### Limitations

Despite a good relationship between the saltiphone output, the loss of mass measured by the balance, and the measured sediment flux by the sediment catchers, there are several limitations for the current reported method. When calibrating saltiphones the output of the instruments should be compared under identical conditions. This is seldom the case, either in a wind tunnel or in the field. In wind tunnels variations in the sediment flux may occur in the test section, such as during our experiments. In the field, spatial and temporal variations in soil roughness, soil moisture content, soil structure and soil texture occur. Also, recalibration or replacement of the microphone is required after some time due to normal wear of the microphones membrane. This was not a problem in the current study but was reported in a previous study [22]. Another problem is that, when the sediment flux is calculated from saltiphone data, shear velocity information is required. This information is usually collected from a vertical tower of anemometers, and thus subject to some uncertainty [44]. Finally, this study used only three types of (industrially washed and sieved) sediment. Although results were very comparable, more tests are recommended, especially with natural sediments characterized by a lower degree of sorting than those used in this study.

## Conclusions and Recommendations

Three samplers were tested in this study: the saltiphone, the MWAC and the BEST. Their efficiencies were tested by comparing the vertically integrated sediment flux measured (or calculated) with these samplers to the emission flux of the sediment source, which was directly measured with a balance. In general, the measured and calculated sediment fluxes are comparable, confirming the usefulness of the samplers and the calculation procedures.

No relationships were found between the efficiency of either sampler and sediment type or wind velocity. Efficiency mainly depends on the design of the samplers, the experimental setup (in particular, the number and elevation of the individual traps in the saltation layer), and on the choice of the regression function when fitting data into the vertical sediment flux or particle velocity profiles.

The saltiphone is a reliable tool to determine aeolian sediment fluxes at fast temporal scales. However, this study was performed in the controlled environment of a wind tunnel. Field conditions are much less stable and usually cannot be controlled, making this type of research much more complicated. However, we think the instrument can produce reliable results under field conditions provided sufficient attention is paid to the experimental setup. Issues to be considered include (but are not limited to): the number of saltiphones in the saltation layer; the vertical distance between adjacent saltiphones (especially close to the bed where sediment transport is highest and the variation of the sediment flux with height is most pronounced); the accuracy in determining the exact elevation of each saltiphone; the difference in sensitivity of each microphone, which affects the acoustic signal; the cleanliness of the output signal, which can be affected by wind or rain; and the measurement interval, which should be identical to the internal sampling rate of the instrument.

When comparing other traps to the saltiphone in the field, attention must also be paid to the distance between the instruments because of very small-scale differences in particle concentration that may occur in the transport layer (sand streamers). Finally, when using the analogue output of the saltiphone to calculate sediment fluxes the wind profile near the bed should be accurately measured, preferably at a sufficiently high temporal resolution.
